# Combining the pan-aurora kinase inhibitor AMG 900 with histone deacetylase inhibitors enhances antitumor activity in prostate cancer

**DOI:** 10.1002/cam4.289

**Published:** 2014-07-03

**Authors:** Channing J Paller, Michel D Wissing, Janet Mendonca, Anup Sharma, Eugene Kim, Hea-Soo Kim, Madeleine S Q Kortenhorst, Stephanie Gerber, Marc Rosen, Faraz Shaikh, Marianna L Zahurak, Michelle A Rudek, Hans Hammers, Charles M Rudin, Michael A Carducci, Sushant K Kachhap

**Affiliations:** 1 Department of Oncology, The Sidney Kimmel Comprehensive Cancer Center, Johns Hopkins Medical InstitutionsBaltimore, Maryland, 21231; 2 Department of Clinical Oncology, Leiden University Medical CenterAlbinusdreef 2, 2333 ZA, Leiden, The Netherlands; 3 Department of Pathology, University Medical Center UtrechtHeidelberglaan 100, 3584 CX, Utrecht, The Netherlands; 4 School of Medicine, Eastern Virginia Medical SchoolNorfolk, Virginia, 23510; 5 Department of Oncology Biostatistics, The Sidney Kimmel Comprehensive Cancer Center, Johns Hopkins Medical InstitutionsBaltimore, Maryland, 21231

**Keywords:** AMG 900, aurora kinase inhibitor, histone deacetylase inhibitors, prostate cancer, synergy, valproic acid, vorinostat

## Abstract

Histone deacetylase inhibitors (HDACIs) are being tested in clinical trials for the treatment of solid tumors. While most studies have focused on the reexpression of silenced tumor suppressor genes, a number of genes/pathways are downregulated by HDACIs. This provides opportunities for combination therapy: agents that further disable these pathways through inhibition of residual gene function are speculated to enhance cell death in combination with HDACIs. A previous study from our group indicated that mitotic checkpoint kinases such as PLK1 and Aurora A are downregulated by HDACIs. We used in vitro and in vivo xenograft models of prostate cancer (PCA) to test whether combination of HDACIs with the pan-aurora kinase inhibitor AMG 900 can synergistically or additively kill PCA cells. AMG 900 and HDACIs synergistically decreased cell proliferation activity and clonogenic survival in DU-145, LNCaP, and PC3 PCA cell lines compared to single-agent treatment. Cellular senescence, polyploidy, and apoptosis was significantly increased in all cell lines after combination treatment. In vivo xenograft studies indicated decreased tumor growth and decreased aurora B kinase activity in mice treated with low-dose AMG 900 and vorinostat compared to either agent alone. Pharmacodynamics was assessed by scoring for phosphorylated histone H3 through immunofluorescence. Our results indicate that combination treatment with low doses of AMG 900 and HDACIs could be a promising therapy for future clinical trials against PCA.

## Introduction

In recent years, the introduction of various novel therapies for prostate cancer (PCA), such as taxanes, has significantly extended survival of patients 1,2. Nevertheless, PCA remains the second deadliest cancer in the Western world 3. Therefore, research is required to further improve clinical outcomes by identifying therapies with improved antitumor efficacy and/or reduced toxicities.

In the search for new molecular targets for PCA treatment, aurora kinases are a promising candidate 4. Three paralogous genes (aurora A, B, and C) comprise the aurora family of serine/threonine protein kinases in mammalian cells. Aurora A and B are essential regulators of mitosis, while aurora C primarily plays a role in meiosis. Aurora A is an oncogene upregulated in several tumor types 5. Its phosphorylation is required for cell cycle progression, centrosome maturation, and spindle assembly 6,7. Aurora B, also overexpressed in tumor cells, is most active during the G2/M-phase, and its phosphorylation is essential for the final steps of cytokinesis 6,8,9. Active aurora B phosphorylates histone H3 on Serine 10, a molecular event vital for chromosome condensation and mitotic progression 6,8. Inhibition of aurora A and B inactivates the spindle assembly checkpoint, resulting in endoreduplication, polyploidy, and eventually, apoptosis 6,10–12. The orally bioavailable pan-aurora kinase inhibitor AMG 900 aborts cytokinesis by inhibition of autophosphorylation of aurora kinases 11. It is effective in multidrug-resistant models, possibly through circumvention of the drug efflux effector P-glycoprotein 11.

AMG 900 may yield enhanced antitumor activity in the presence of additional cancer therapeutics 11,13. Previous studies by our group have demonstrated that several genes involved in mitotic checkpoints, including polo-like kinase 1 (Plk1) and aurora kinases, are downregulated by HDACIs 14,15. Recently, we have demonstrated that combination of HDACIs with a Plk1 inhibitor synergistically induced apoptosis, decreased cell proliferation, and decreased clonogenic survival of PCA cells 16. Based on our success with Plk1 inhibitors, we hypothesized that addition of HDACIs could potentiate apoptosis in PCA cells that are treated with Aurora kinase inhibitors. Further, HDACIs exhibit promising antitumor effects in PCA in vitro and in vivo 17,18, and have successfully been used in concert with other chemotherapeutics 19,20. Therefore, HDACIs could serve as a rational choice for complementing the apoptotic effects of AMG 900. Hence, we combined AMG 900 with the HDACIs VPA and vorinostat in PCA cells in our current study 15. We found that combination of HDACIs with AMG 900 has a synergistic antitumor effect, the HDACIs activating an apoptotic mechanism in aurora kinase-inhibited PCA cells.

## Material and Methods

### In vitro

#### Cell culture and treatment

PCA cell lines (DU-145, LNCaP, PC3) were obtained from ATCC. Cells were grown in RPMI-1640 (Invitrogen, Carlsbad, CA) with 10% fetal bovine serum (FBS) (Gemini, West Sacramento, CA) and maintained in a 37°C humidified incubator supplemented with 5% CO_2_. VPA (Sigma-Aldrich, St Louis, MO) was prepared in Roswell Park Memorial Institute (RPMI) at a 1 mol/L stock on the day of treatment of the cells. Vorinostat (AtonPharma, Lawrenceville, NJ) and AMG 900 (Amgen, Thousand Oaks, CA) were maintained in 10 mmol/L dimethyl sulfoxide (DMSO) stock solutions at −20°C and diluted in RPMI upon use. Compounds were administered concomitantly in combination studies.

#### Cell viability and synergy

3-(4,5-Dimethylthiazol-2-yl)-5-(3-carboxymethoxyphenyl)-2-(4-sulfophenyl)-2H-tetrazolium (MTS) assays were performed with CellTiter 96™ Aqueous Nonradioactive Cell Proliferation Assay reagent (Promega, Madison, WI) according to the manufacturer's instructions. In brief, PCA cells were plated in 96-well plates, allowed to adhere overnight and treated with the selected compounds for 72 h. Subsequently, MTS reagent was added. Absorption at 490 nm was measured after approximately 2 h using a colorimetric plate reader (Molecular Devices, Sunnyvale, CA).

To compare the antitumor effect of single-agent treatments with combination treatment, synergy was determined using CalcuSyn software (Biosoft, Cambridge, U.K.). CalcuSyn calculates a combination index (CI) at different levels of growth, using the formula for mutually nonexclusive mechanisms: (D1/Dx1) + (D2/Dx2) + (D1 × D2/Dx1 × Dx2), where D1 and D2 are the doses of drug 1 and drug 2 in combination required to produce × percentage effect, and Dx1 and Dx2 are the doses of drug 1 and drug 2 alone required to produce the same effect. Synergy levels (no synergy [CI > 0.9], moderate synergy [0.7 < CI < 0.9, +], synergy [0.3 < CI < 0.7, ++], strong synergy [0.1 < CI < 0.3, +++], very strong synergy [CI < 0.1, ++++]) were determined from CI ranges, using the Chou–Talalay method following the manufacturer's instructions 21,22.

#### Cell survival

Clonogenic assays were performed to assess long-term cell survival. PCA cells were plated in complete RPMI media. Upon reaching 50–60% confluency, drugs were added at the appropriate concentration and dishes were incubated for 48 h. Then cells (1.25 × 10^3^ for DU-145 and PC3 cells, 2 × 10^3^ for LNCaP cells) were replated and grown in triplicate in 60 mm dishes containing fresh, complete RPMI media. After 10–14 days (depending on the doubling time of cell line), crystal violet stain (Sigma) was used to stain colonies and colonies were counted. All dishes from one cell line were stained at the same time point. The average number of colonies in DMSO-treated controls was considered 100% clonogenic survival in each separate cell line and in each separate experiment. Student's *t*-tests were performed to assess whether clonogenic survival of a cell line differed significantly between doses of a single agent; synergy was determined with CalcuSyn when comparing single-agent treatment with combination treatment 21,22.

#### Cellular senescence

PCA cells were plated in 6-well plates (25–50 × 10^3^ cells per well) and allowed to adhere overnight. Compounds were added to the complete RPMI media for 48 h, after which senescent cells were stained using the senescence *β*-galactosidase-staining kit (Cell Signaling Technology, Danvers, MA) according to the manufacturer's instructions. In brief, cells were washed in phosphate buffered saline (PBS) and fixed in Fixative Solution (2% formaldehyde and 0.2% glutaraldehyde in 1× PBS). After fixing, cells were washed in PBS and incubated with Staining Solution (containing 40 mmol/L citric acid/sodium phosphate [pH 6.0], 150 mmol/L NaCl, 2 mmol/L MgCl_2_, 5 nmol/L potassium ferrocyanide, and 1 mg/mL X-gal in 5% dimethylformamide) in a 37°C incubator for 24 h. Cells were washed in PBS and visualized under an Olympus IX70 inverted microscope (Tokyo, Japan) using an Uplan FL 10× phase contrast lens. Multiple images (>5) were taken from each well. Senescent (blue) and total cells were counted in five fields of approximately 30 cells/field for each treatment. Student's *t*-tests were performed to determine whether cellular senescence significantly differed between combination treatment and single-agent treatment.

#### Fluorescence microscopy

Cells were drugged at 50–70% confluency for 48 h and fixed with neutral-buffered formalin for 10 min, followed by permeabilization with 0.125% Triton X-100 for 5 min. Cells were blocked with 10% bovine serum albumin (BSA) overnight. Cells were probed with a primary antibody against phosphorylated aurora kinase A/B/C (Antibody #2914; Cell Signaling Technology) at a 1:100 dilution followed by an Alexa Fluor-555 (Invitrogen) conjugated secondary antibody at a 1:400 dilution in blocking buffer. Cells were washed in PBS and further incubated with Alexa Fluor-488 conjugated phosphorylated histone H3 antibody (antibody #9713 1:100 dilution; Cell Signaling Technology). Cells were counterstained with Hoechst 33258 and mounted on slides. Confocal images were taken with the Zeiss LSM 510 meta-confocal microscope (Carl Zeiss, Thornwood, NY) using a 63× objective.

#### Flow cytometry

Cells were plated in 100 mm dishes and drugged at 50–70% confluency. Both floating and attached cells were collected 48 h after treatment, washed in PBS and fixed with 4% freshly made paraformaldehyde. Cells were then permeabilized with 90% cold methanol. Permeabilized cells were stained with Alexa Fluor-488 conjugated phosphorylated histone H3 antibody (1: 100 dilution; Cell Signaling Technology). Nuclei were stained with propidium iodide (Sigma) in PBS containing 1% BSA. Flow cytometry was performed on a FACSCalibur flow cytometer (BD Biosciences, San Jose, CA). Cell cycle analysis was performed using BD FACSDiva software and FlowJo.

#### Immunoblotting

Western blotting was performed as described previously 14. Loading volumes equal to 20 *μ*g of total protein were used. Primary antibodies were diluted 1:1000 in blocking solution with the exceptions of vinculin (Millipore, Billerica, MA) and phosphorylated aurora A/B/C (Antibody #2914; Cell Signaling Technology), which were diluted 1:4000 and 1:500, respectively. Conjugated secondary antibodies were diluted 1:4000 in blocking solution. Blocking solution (5% milk in TBST (10[mmol/L Tris-HCl pH 7.4, 0.1% Tween 20, 150 mmol/L: NaCl in H_2_O]) was used to dilute antibodies against p21 (BD Biosciences), cyclin B1 (Cell Signaling Technology), vinculin, and cleaved PARP (Cell Signaling Technology), as well as all secondary antibodies. 5% BSA was used for phosphorylated aurora A/B/C and phosphorylated and total histone H3 (Cell Signaling Technology). Blots were developed with enhanced chemiluminescence (ECL) (GE) or Femto (Pierce Biotechnology, Rockford, IL) and scanned into a computer at a resolution of 300 dots per inch (dpi). Densitometric analyses were performed using ImageJ (Research Services Branch, National Institute of Mental Health); density of bands of the protein of interest was normalized to the density of bands of the housekeeper (actin, vinculin or total aurora A), and protein expression of treated cells was compared to the control.

### In vivo

#### Animals

The animal protocol was approved by the Institutional Animal Care and Use Committee (IACUC) of Johns Hopkins University. All IACUC guidelines and United States Department of Agriculture regulations were followed. The mice used in this study were 8-week old JHU Oncology nonobese diabetic (NOD)/severe combined immunodeficiency (SCIDs) (JHU bred colony) and were housed under aseptic conditions on a 12-h light–dark cycle with food and water provided ad lib. Each cage contained ≤5 mice, which were differentiated by treatment groups.

Two million DU-145 PCA cells, suspended in complete RPMI media, were embedded in a 1:2 solution of Matrigel (BD Biosciences) and injected subcutaneously into the right flank of the mice. The tumor inoculation success rate was approximately 90%. Following a 3-week growth incubation period, tumor volume was estimated with digital calipers, using the standard formula: *π*/6 × length × width × height. Before treatment initiation, mice were stratified by tumor size and assigned into homogenous groups (8–9 per group). Once average tumor volume was above 200 mm^3^, treatment was initiated in all mice. Treatments were administered four consecutive days per week for a total duration of 4 weeks. Vorinostat (50 mg/kg) was administered once daily via intraperitoneal injections on mornings of days 1–4 of the dosing cycle. AMG 900 was administered through gavage on days 1 and 2 of each dosing cycle. Mice were treated with vehicle (2% hydroxylpropyl methyl cellulose [HPMC] and 1% Tween 80 in deionized water [pH 2.2] with methane sulfonic acid [MSA]), or AMG 900 at a concentration of 3.75 mg/kg or 7.5 mg/kg (provided weekly by Amgen in glycerin). Tumor size and mice bodyweights were measured on the day preceding each dosing cycle (day 0/7) and the final day of dosing (day 4) each week. Tumors were harvested when the volume reached 1000 mm^3^, which occurred 26–35 days after start of the treatment. For histological assessment of the tumors, mice were perfused with 2% paraformaldehyde through a cardiac catheter, and then tumors were excised, infiltrated in sucrose, embedded in optimum cutting temperature (O.C.T.) compound (Sakura Finetek, Tokyo, Japan), and stored at −80°C. Tissue sections were prepared for hematoxylin–eosin staining by fixing the tissue in formalin and embedding in paraffin after tumor excision.

#### Tumor growth analysis

In vivo data from the DU-145 xenograft model were analyzed with a random intercept hierarchical linear model. The primary statistical outcome was tumor volume. To adjust for the initial volume, tumor volumes on days 3 through 26 for each mouse were divided by the volume on day 0 and then the log of these values was taken for analysis. The intercept in this longitudinal model was specified such that it represented the log ratio of the final tumor volume to the initial volume (time was coded using negative numbers and 0 for the final day). The model had the formula:


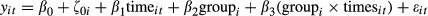


where *y*_*it*_ denotes the log ratio tumor volume for mouse *i* at time *t*, *β*_0_ is the intercept representing the log ratio of the tumor volume on day 26 to the initial volume for the control group, *β*_1_ is the linear effect of time for the control group, *β*_2_ is the group effect on day 26, and *β*_3_ is the group effect in terms of the linear effect of time. The mouse-specific effect *ζ*_0*i*_ represented the deviation of each mouse from the group intercept. This is corrected for the correlation between measurements taken on the same mouse. Since the mice in this experiment were considered a representative sample from a larger population, the effect was considered random and it was assumed that the population distribution from which they were sampled had a normal distribution.

#### Tissue immunostaining

Sections (20 *μ*m) were cut from frozen tissues and mounted on microscope slides (Fisher Scientific, Waltham, MA). Sections were blocked in blocking buffer (5% goat serum, 0.5% BSA, 0.1% Triton X-100 and 0.01% sodium azide in PBS) and probed overnight with the primary antibody phosphorylated histone H3 in a 1:100 dilution (antibody #9713; Cell Signaling Technology). Sections were washed in PBS containing 0.1% Triton X-100 and probed overnight with Alexa Fluor-546 conjugated anti-rabbit antibody in a 1:500 dilution (Invitrogen). Subsequently, sections were washed in PBS and fixed with 10% formalin, and nuclei were stained with DAPI (4′,6-diamidino-2-phenylindole) present in the mountant (Invitrogen). Coverslips were mounted on the slides and sections were imaged with a Nikon Eclipse Ti microscope at 20× (Nikon, Tokyo, Japan). The percentage of fluorescent cells was assessed in at least three fields of view per treatment group by dividing the density of red (phosphorylated histone H3 positive cells) by the density of blue (DAPI staining all nuclei) staining with ImageJ. Student's *t*-tests were performed to assess for statistically significant differences in the percentage of cells that stained positively for phosphorylated histone H3 and phosphorylated aurora A/B/C.

## Results

### AMG 900 and HDACIs inhibit aurora kinase expression and clonogenic survival of PCA cells

**Figure 1:**
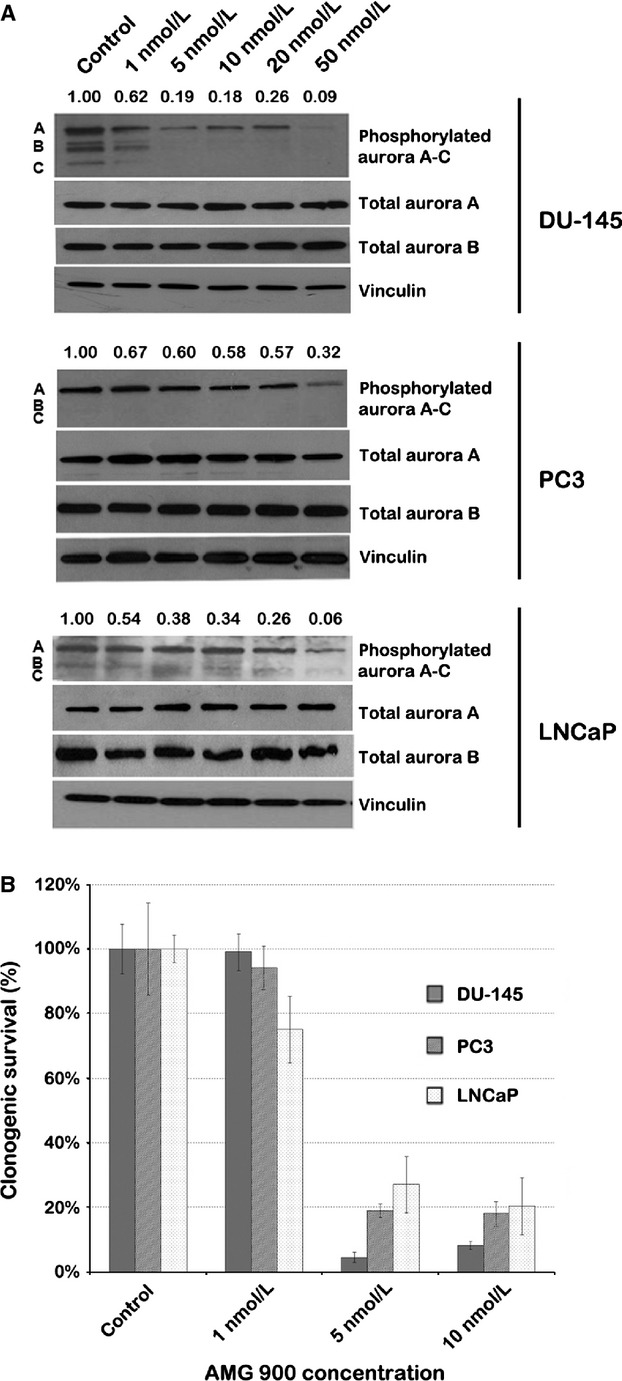
AMG 900 effectively targets PCA cells at concentrations above 1 nmol/L. (A) Western blot quantifying the protein levels of phosphorylated aurora A (48 kDa), B (40 kDa) and C (35 kDa) after treatment of PCA cells with AMG 900 (concentrations indicated above each lane) for 48 h. Bands were normalized to total levels of aurora A. (B) Bar graph representing relative clonogenic survival after AMG 900 treatment of PCA cell lines. Colonies were counted in triplicate. PCA, prostate cancer.

After treatment of DU-145, PC3 and LNCaP PCA cells with AMG 900 for 12 h, the relative decrease in protein expression of phosphorylated aurora A/B/C was compared to protein levels of total aurora A. In all three cell lines, protein expression of phosphorylated aurora decreased in a dose-dependent manner at concentrations above 1 nmol/L (Fig.1A). Next, long-term clonogenic assays were performed to determine cell survival of PCA cells after AMG 900 treatment at 1 and 5 nmol/L. At 5 nmol/L, AMG 900 effectively inhibited clonogenic survival in all PCA cell lines (>70%) (Fig.1B). These results mirrored those of Payton et al., who reported that the IC_50_ of AMG 900 in proliferation assays was around 5 nmol/L for DU-145 and PC3 cells 11. Based on aforementioned results, we selected AMG 900 at 1 nmol/L (low dose, less effective) and 5 nmol/L (high dose, effective) for drug combination studies. VPA and vorinostat were administered at concentrations of 1 mmol/L and 1.5 mmol/L, and 0.5 *μ*mol/L and 1 *μ*mol/L, respectively, as at these concentrations the compounds downregulated phosphorylated aurora A/B/C protein expression levels in DU-145 and PC3 cells (Fig. S1), while previous studies suggested acceptable toxicities at these concentrations 16,18,23.

### Combinations of low-dose AMG 900 with HDACIs decrease proliferation activity and clonogenic survival of PCA cells

**Figure 2:**
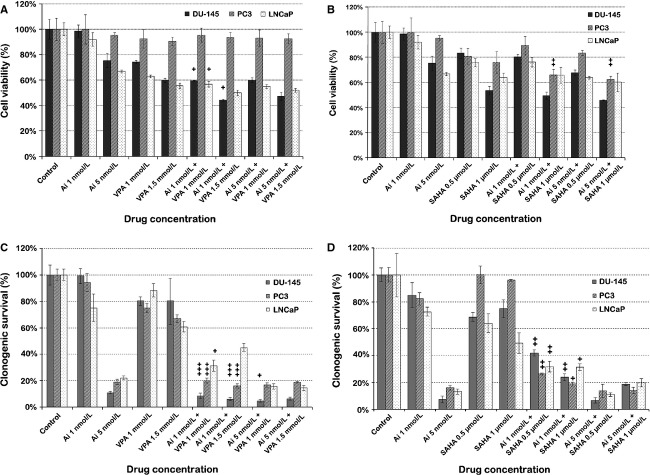
Combinations of AMG 900 with HDACIs VPA and vorinostat decrease the proliferation activity and long-term clonogenic survival of PCA cells compared to single-agent use. (A and B) Proliferation activity of PCA cells after treatment with AMG 900 and VPA (A) or vorinostat (B), as measured by MTS assays. (C and D) Quantification of colonies to assess clonogenic survival of PCA cells after treatment with AMG 900 and VPA (C) or vorinostat (D). Ai, AMG 900; +, moderate synergy; ++, synergy; +++, strong synergy; HDACI, histone deacetylase inhibitors; PCA, prostate cancer; SAHA, suberanilohydroxamic acid (vorinostat); VPA, valproic acid.

We employed MTS and clonogenic assays to assess the effect of combinations of AMG 900 with VPA and vorinostat on the proliferation activity and long-term survival of PCA cells compared to single agent. In both assays, treatment of PCA cells with 1 nmol/L AMG 900 did not result in antitumor activity, similar to previous results (compare Fig.1 to Fig.2). In MTS assays, low-dose VPA combined with low-dose AMG 900 showed enhanced inhibition of cell proliferation compared to high-dose AMG 900 used as a single agent in both DU-145 and LNCaP cells (Fig.2A). Moderate synergistic effects were observed in DU-145 cells treated with combinations of AMG 900 (1 nmol/L) and VPA (1 mmol/L and 1.5 mmol/L) (CI = 0.796 and CI = 0.777, respectively), and in LNCaP cells treated with combinations of AMG 900 (1 nmol/L) and VPA (1 mmol/L) (CI = 0.848) (Table S1). In PC3 cells the proliferation, as evaluated by MTS assays, was decreased by 10% at the most after treatment with AMG 900 and/or VPA. Combinations of vorinostat and AMG 900 enhanced the inhibition of cell proliferation in all three cell lines compared to treatment with single agents, except when combining a low dose of vorinostat (0.5 *μ*mol/L) with a low-dose of AMG 900 (1 nmol/L) (Fig.2B). Synergistic effects, as defined by CalcuSyn, were observed in PC3 cells treated with combinations of 1 *μ*mol/L vorinostat and AMG 900 (1 nmol/L and 5 nmol/L) (CI = 0.375 and CI = 0.558, respectively) (Table S1) 21,22.

Cell death through aurora kinase inhibition may involve aborted cytokinesis progressing to apoptosis, which may not be registered as a change in a short-term proliferation assay. Therefore, we assessed the long-term effect of combination treatment on clonogenic survival in PCA cell lines. Treatment of PCA cells with 1 nmol/L AMG 900 did not result in decreased clonogenic survival; however, combinations of low-dose AMG 900 with either VPA or vorinostat resulted in a remarkably decreased clonogenic survival compared to single-agent treatment (Fig.2C and D). Subsequent analyses performed with CalcuSyn demonstrated that administration of AMG 900 (1 nmol/L) combined with VPA (1 mmol/L and 1.5 mmol/L) resulted in a strongly synergistic decrease in survival compared to the single compounds in DU-145 and PC3 cells (0.171 < CI < 0.260) (Fig.2C, Table S1) 21,22. In LNCaP cells a moderately synergistic decrease in clonogenic survival was seen in combination treatments of low-dose AMG 900 and low-dose VPA (CI = 0.765). Treatment of cells with combinations of AMG 900 (1 nmol/L) and vorinostat (0.5 and 1 *μ*mol/L) resulted in a decrease in clonogenic survival that was synergistic or moderately synergistic in all three PCA cell lines (0.340 < CI < 0.809) (Fig.2D, Table S1). The higher dose of AMG 900 as a single agent severely inhibited clonogenic survival in all three cell lines. As a result, virtually no synergy could be measured in combination treatments involving high-dose AMG 900.

### Combinations of AMG 900 with VPA or vorinostat increase cellular senescence of PCA cells

**Figure 3:**
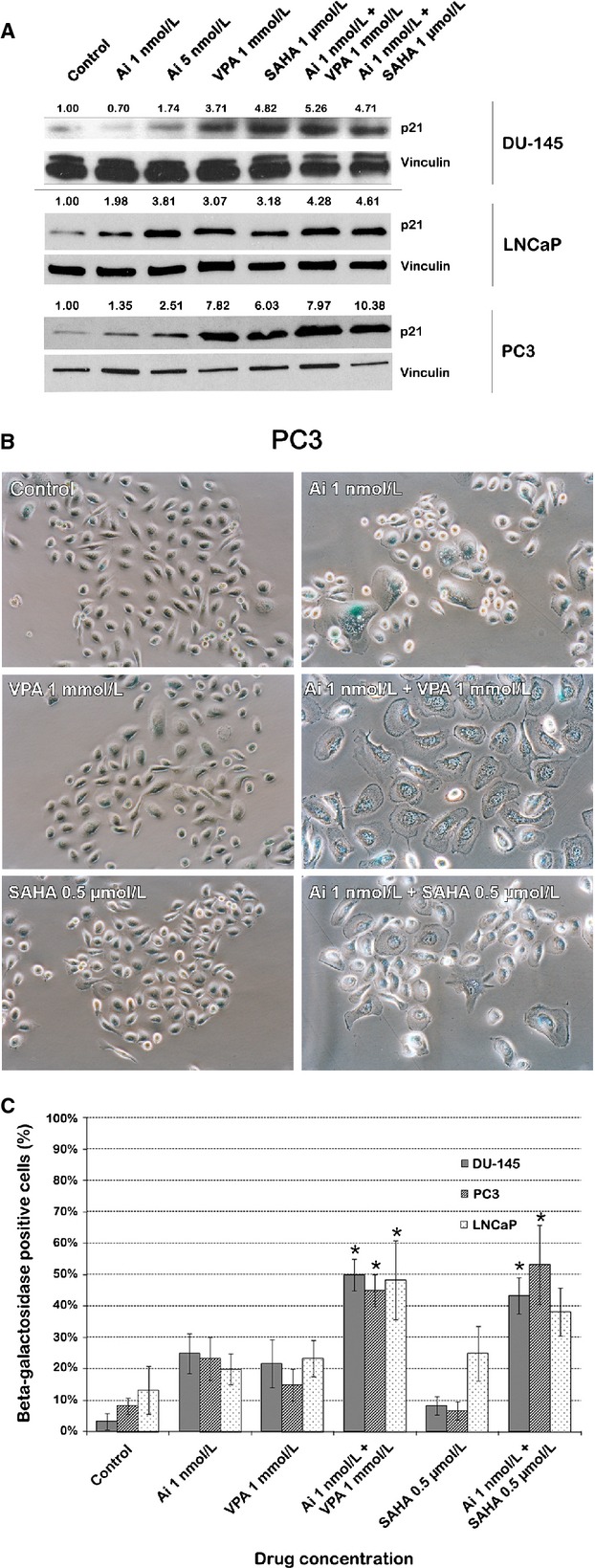
AMG 900 combined with HDACIs VPA or vorinostat increases cellular senescence in PCA cell lines compared to single-agent use. (A) Western blot for p21, a marker of cellular senescence or a G1/G2 phase cell cycle arrest, after treating DU-145, LNCaP and PC3 cells as indicated above each lane. Bands were normalized to the housekeeper vinculin. Ai, AMG 900. (B) Representative images of PC3 cells after performing an SA *β*-galactosidase assay. Blue cells are SA *β*-galactosidase positive cells, indicating cellular senescence. Ai, AMG 900. (C) Quantification of SA *β*-galactosidase-positive staining after treatment of PCA cells with AMG 900 and/or HDACIs VPA/vorinostat. Ai, AMG 900; * combination treatments with a significantly increased percentage of senescent cells (*P* ≤ 0.05). HDACI, histone deacetylase inhibitors; PCA, prostate cancer; SAHA, suberanilohydroxamic acid (vorinostat); VPA, valproic acid.

During MTS and clonogenic assays, we observed PCA cell phenotypes resembling a senescent morphology after treatment with AMG 900 with or without HDACIs. Treated cells displayed a flattened morphology compared to untreated controls, and some treated DU-145 and PC3 cells showed cytoplasmic vacuoles and granularity. We performed a Western blot analysis for p21 as a surrogate marker of cellular senescence in PCA cells treated with (combinations of) AMG 900 and HDACIs 24. Protein levels of p21 were not assessed in combinations with high-dose AMG 900, as during the course of the treatment, cells treated with this combination constituted mainly apoptotic cells confounding analysis. In line with previous studies 18,25, protein expression of p21 was significantly increased after HDACI treatment compared to untreated controls; to a lesser extent AMG 900 treatment also resulted in an increase in p21 protein levels (Fig.3A). Combination treatment further increased p21 levels, suggesting increased cellular senescence. To ascertain whether cellular senescence is indeed increased after combination treatment compared to single-agent use in PCA cells, we stained DU-145, PC3 and LNCaP cells for senescence-associated *β*-galactosidase (SA *β*-gal) enzyme activity (Figs.3B and S2). Consistent with our previously observed morphological transformations, DU-145 and PC3 cells treated with combinations of AMG 900 and HDACIs had increased SA *β*-galactosidase staining. LNCaP cells had a baseline level of blue SA *β*-galactosidase staining in untreated controls; combination treatment of LNCaP cells with AMG 900 and HDACIs resulted in increased SA *β*-galactosidase staining. Quantification of *β*-galactosidase-positive cells confirmed that the percentage of senescent cells was significantly increased in PCA cells treated with combinations of AMG 900 and HDACIs (35–60% SA *β*-galactosidase positive cells) compared to cells treated with single agents alone (≤25% SA *β*-galactosidase positive cells) (*P* ≤ 0.05), except for combination treatment of LNCaP cells with AMG 900 (1 nmol/L) and vorinostat (0.5 *μ*mol/L) compared to cells treated with vorinostat alone (*P* = 0.116) (Fig.3C).

### Combination treatment with AMG 900 and HDACIs increases PCA cells with multipolar spindles and polyploidy as compared to single-agent treatment

**Figure 4:**
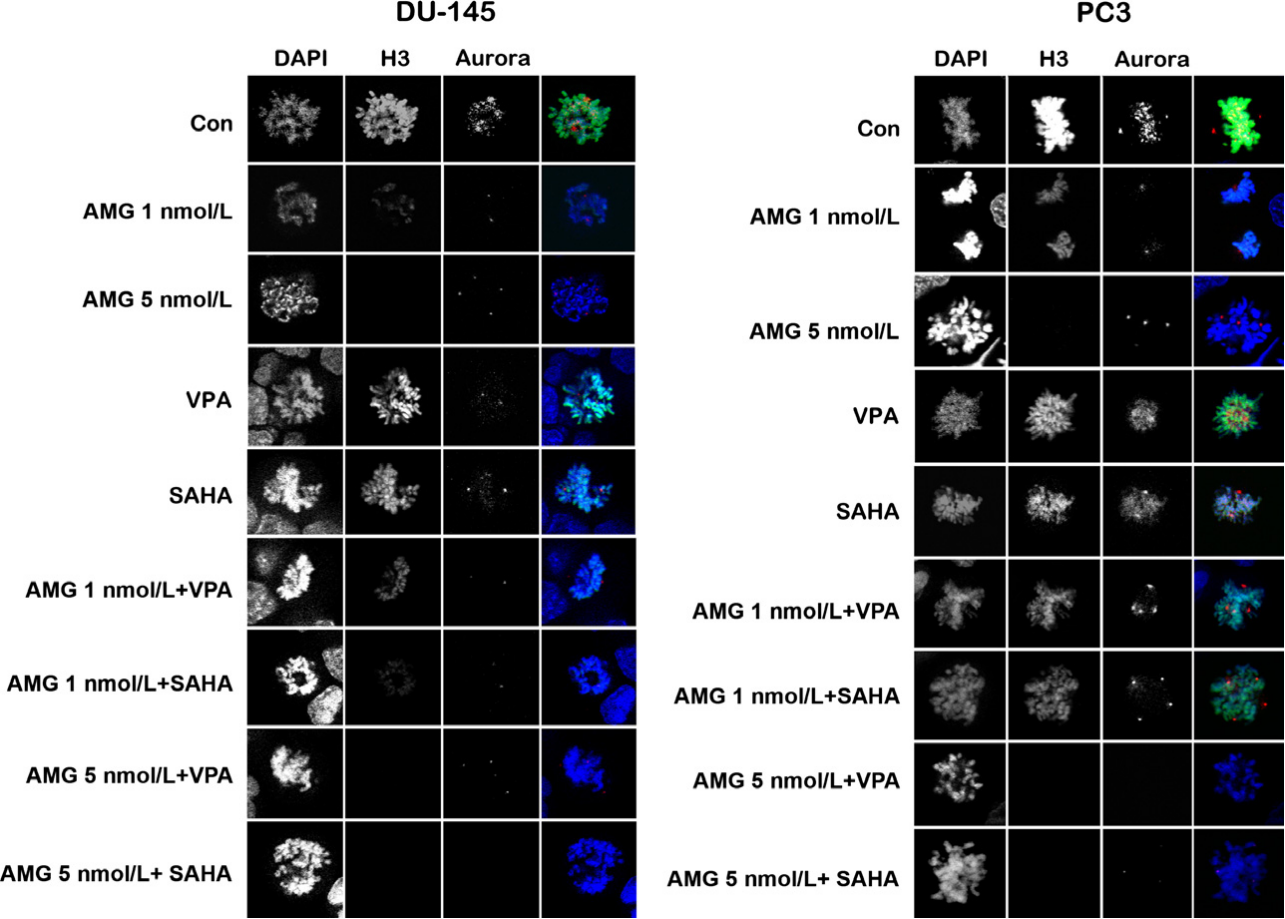
Confocal analysis of DU-145 and PC3 PCA cells treated with AMG 900 (1 or 5 nmol/L) and HDACIs VPA (1 mmol/L) and vorinostat (1 *μ*mol/L) stained for phosphorylated histone H3 (green) and phosphorylated aurora kinase A/B/C (red). AMG 900 causes a decrease in phosphorylated histone H3 and an increase in multipolar spindles in a dose-dependent manner. PC3 cells also demonstrate multipolar spindles when treated with a combination of lower concentrations of AMG 900 and HDACIs. Ai, AMG 900; HDACI, histone deacetylase inhibitors; SAHA, suberanilohydroxamic acid (vorinostat); VPA, valproic acid.

Since aurora kinases localize to distinct subcellular structures in mitotic cells, we probed DU-145 and PC3 cells with an antibody against phosphorylated aurora kinase A/B/C to investigate whether treatment results in differences in localization of these enzymes (Fig.4). We costained the cells for phosphorylated histone H3 as histone H3 is phosphorylated at Ser 10 by aurora B during mitosis and can therefore be used as a marker for aurora B activity 26. After treatment with AMG 900, alone or in combination with HDACIs, localization of aurora was limited to the spindle poles. It was further observed that PC3 cells treated with low-dose AMG 900 and HDACIs exhibited multipolar spindles, similar to 5 nmol/L of AMG 900, suggesting endoreduplication and polyploidy. Both cell lines showed a near complete loss in aurora staining after a combination treatment with 5 nmol/L of AMG 900 and HDACIs. Of note, addition of AMG 900 also caused a decrease in histone H3 phosphorylation in a dose-dependent manner in both PCA cell lines, with DU-145 demonstrating a greater decrease compared to PC3 cells. These results demonstrate that AMG 900 inhibits aurora kinases in PCA cells, potentially causing an increase in multipolar polyploid cells.

**Figure 5:**
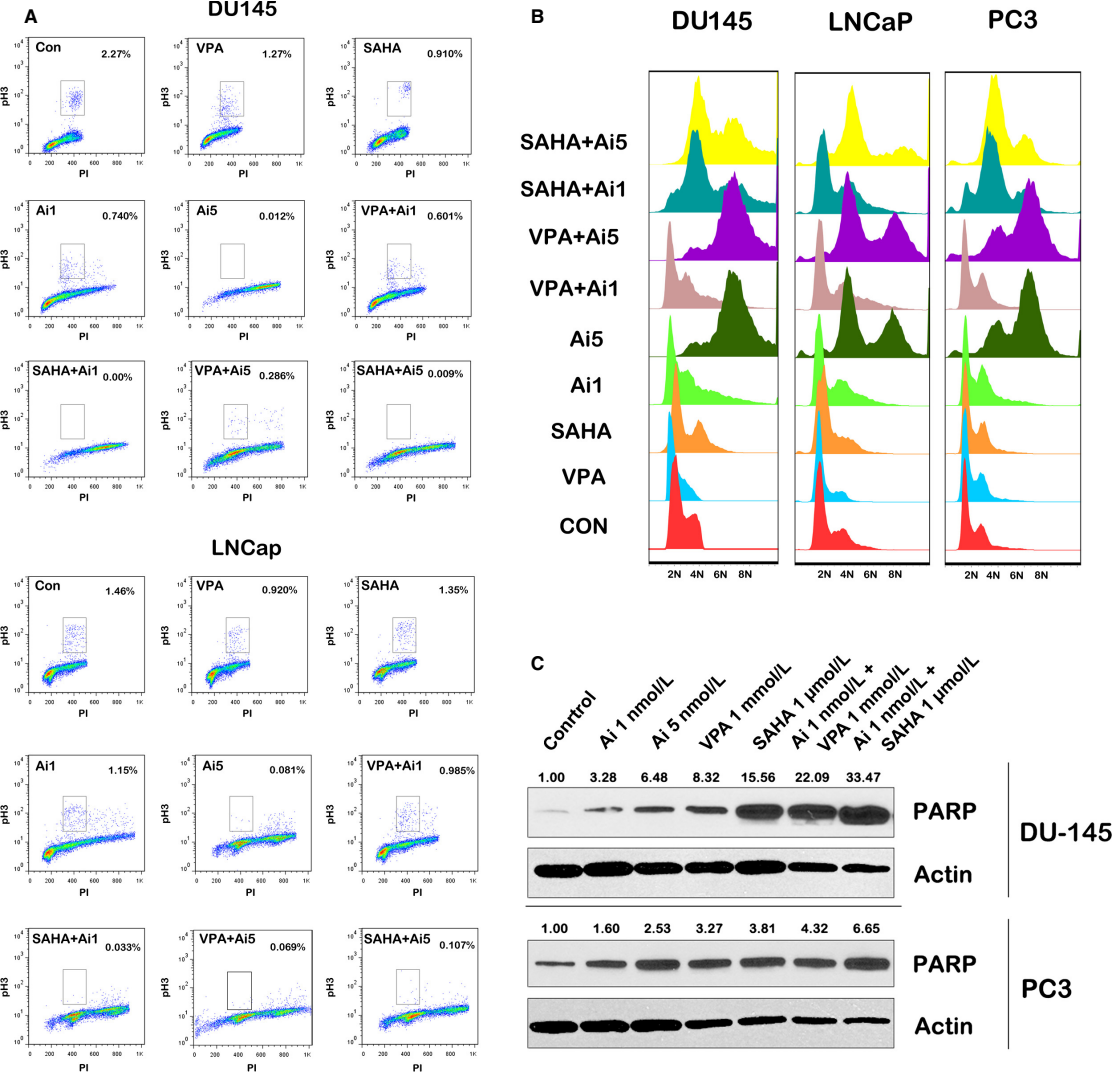
Flow cytometry analysis of PCA cell lines treated with AMG 900 (1 or 5 nmol/L) and HDACIs VPA (1 mmol/L) and vorinostat (1 *μ*mol/L). (A) Scatter plots show a decrease in phosphorylated histone H3 in PCA cells treated with AMG 900 in a dose-dependent manner. Cells treated with a combination of vorinostat and low-dose AMG 900 show a marked decrease in phosphorylated histone H3 as compared to single-agent treatment. (B) Cell cycle analysis indicates that AMG 900 causes an increase in polyploidy in all PCA cell lines in a dose-dependent manner. Low-dose combination of AMG 900 with HDACIs cause an increase in polyploidy as compared to single-agent treatment. Ai, AMG 900. (C) Western blot analysis for cleaved PARP in PCA cells treated with AMG 900 and/or HDACIs (the treatment is indicated above each lane). Bands were normalized to the housekeeper actin. Ai, AMG 900; HDACI, histone deacetylase inhibitors; PCA, prostate cancer; SAHA, suberanilohydroxamic acid (vorinostat).

To further quantify the effects of combining HDACIs and AMG 900 in PCA cells, we performed cell-cycle analysis after treatment (Fig.5A and B). Cells were stained with phosphorylated H3 as a marker of aurora kinase activity. It is also a marker for cells in mitosis. DU-145 cells exhibited the greatest dose-dependent decrease in phosphorylated histone H3 positive cells upon AMG 900 treatment (Fig.5A). In both cell lines, combination treatment resulted in an additional decrease in phosphorylated histone H3 positive cells, most evidently after combination therapy with the HDACI vorinostat. As expected, flow cytometry showed AMG 900 alone induced polyploidy in a dose-dependent manner with DU-145 cells exhibiting increased polyploidy as compared to LNCaP and PC3 (Fig.5B). Combinations of vorinostat and low-dose AMG 900, again to a greater extent than VPA and low-dose AMG 900, led to a much greater increase in polyploidy as compared to single agents across PCA cell lines investigated.

All above data pointed to enhanced antitumor effects after treatment with a combination of low concentration of AMG 900 with HDACIs. To investigate whether combinations led to increased apoptosis, we probed PCA cells for cleaved PARP, a marker for apoptosis. As expected, combination treatments with low-dose AMG 900 resulted in an increase in PARP cleavage (Fig.5C). DU-145 cells exhibited a greater increase in PARP cleavage as compared to PC3 cells. These data demonstrate that AMG 900 can effectively inhibit aurora kinase activity in PCA cells, although the degree of inhibition may differ between cell types. Addition of an HDACI further potentiates apoptosis.

### Low-dose AMG 900 in combination with vorinostat inhibits histone H3 phosphorylation and suppresses growth of DU-145 xenografts

**Figure 6:**
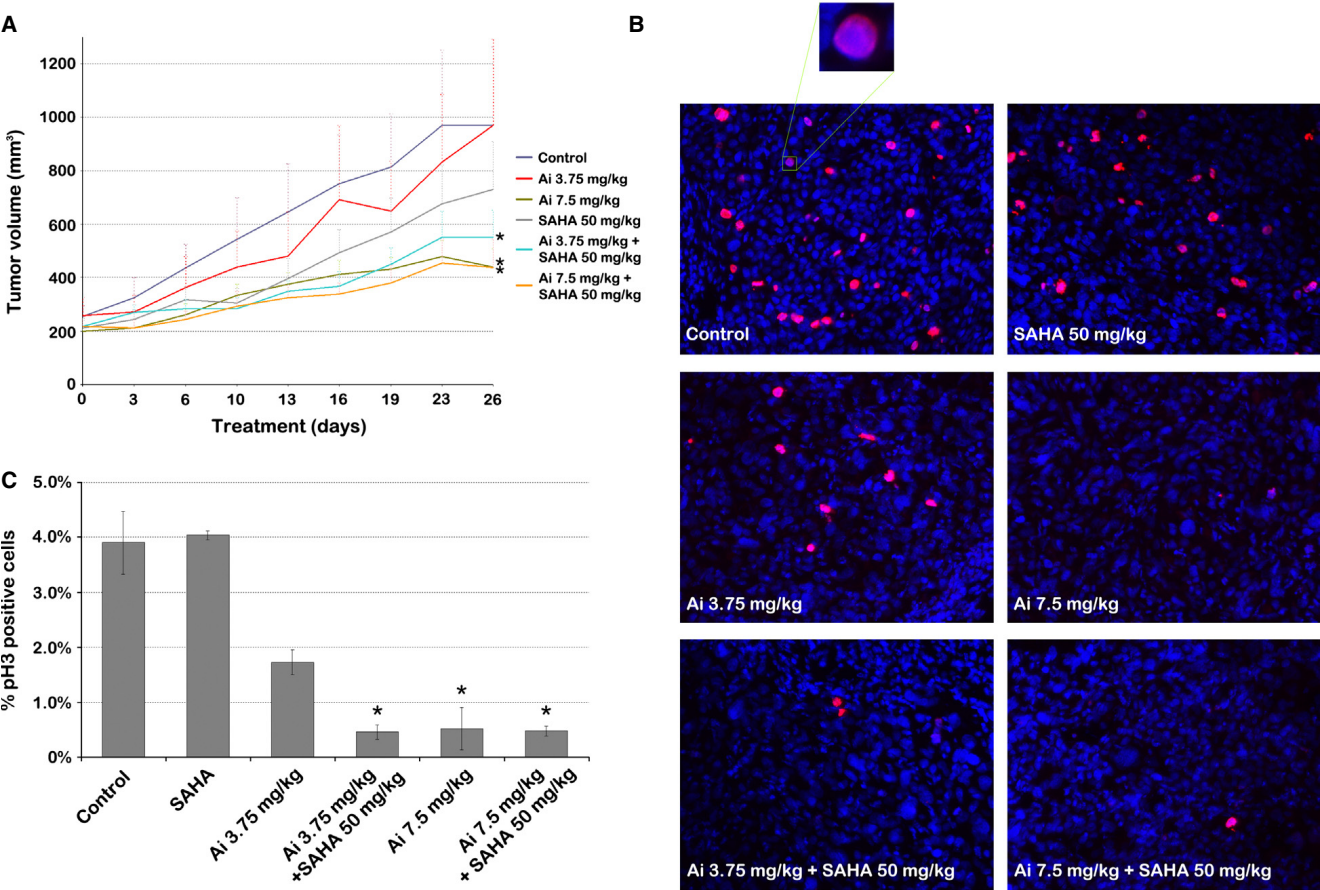
Low-dose AMG 900 in combination with vorinostat enhances growth suppression of DU-145 xenografts and inhibits histone H3 phosphorylation in vivo, similar to treatment with high-dose AMG 900. (A) Tumor growth curves of NOD/SCID mice bearing established DU-145 tumors treated with vehicle alone, AMG 900 and/or vorinostat for a maximum of 4 weeks. Tumor volumes are represented as mean ± SEM (*n* = 9). *treatment groups that had a significantly different tumor growth rate compared to the vehicle-treated group (*P* ≤ 0.05). (B) Representative images of tumor sections stained with phosphorylated histone H3 (red) and DAPI (blue) indicate decreased phosphorylated histone H3-positive cells after AMG 900 treatment and combination treatment. (C) Bar graph quantifying the percentage of phosphorylated histone H3-positive cells per treatment group. Student's *t*-tests confirm significantly decreased phosphorylated histone H3 staining after high-dose AMG 900 treatment and after combination treatment. *treatment groups that had significantly less phosphorylated histone H3-positive cells compared to the vehicle-treated group (*P* ≤ 0.05). Ai, AMG 900; NOD, nonobese diabetic; pH3, phosphorylated histone H3; SAHA, suberanilohydroxamic acid (vorinostat); SCID, severe combined immunodeficiency.

The effect of combination treatment with AMG 900 and HDACIs on in vivo tumor growth inhibition was evaluated in mice bearing DU-145 xenografts. Vorinostat was selected as HDACI in these experiments, as it is already approved for the treatment of cancers in humans and the treatment administration does not require continuous administration as does VPA in mice 18. Mice were treated with vehicle alone, with AMG 900 at 3.75 (low dose) or 7.5 mg/kg (high dose), and/or with vorinostat at 50 mg/kg, corresponding to doses that had been used previously with limited toxicity 11,27. The mean tumor growth during treatment is depicted in Figure6A. We used the random intercept model to assess the overall effects for time, group, and the time by group interaction, which were all significant (interaction *P* < 0.0001, for details please refer to Data S1). Specifically, the tumor growth rate in mice after single-agent treatment with high-dose AMG 900 and combination treatment with either low- or high-dose AMG 900 and vorinostat was significantly reduced compared to the tumor growth rate in control mice (*P*-values 0.020, 0.014, and 0.036, respectively) (Tables S3–S5). In the group treated with a combination of low-dose AMG 900 and vorinostat the average tumor growth rate was also lower than the tumor growth rate in groups treated with low-dose AMG 900 alone or vorinostat alone (*P* = 0.003 and *P* = 0.008, respectively). Tumor growth rates in mice treated with low-dose AMG 900 and vorinostat combination treatment were similar to tumor growth rates in mice treated with high-dose AMG 900 (*P* = 0.833) and in mice treated with high-dose AMG 900 and vorinostat (*P* = 0.721).

These data are in concordance with our in vitro findings that the combination of low-dose AMG 900 and HDACIs enhances inhibition of tumor cell growth. As the groups receiving combination treatment did not exhibit a significant difference in overall bodyweight compared to vehicle-treated controls and did not show signs of severe toxicity (no diarrhea or lethargy), our data suggest that combination treatment resulted in few toxicities in mice with stable weight (Tables S6 and S7, Figs. S5–S7).

Tumors from sacrificed mice were stained for phosphorylated histone H3 to assess aurora B inhibition (Fig.6B). Vehicle- and vorinostat-treated tumors displayed about 4% phosphorylated histone H3 positive staining (*P* = 0.713), while treatment with low-dose AMG 900 decreased the percentage of phosphorylated histone H3 positive cells to about 2% (*P* = 0.004) (Fig.6C). Both combination treatment with low-dose AMG 900 and vorinostat, and treatment with high-dose AMG 900 alone or in combination with vorinostat resulted in 0.5% of the DU-145 cells being stained positively for phosphorylated histone H3, indicating significantly inhibited aurora B kinase activity in these tumors compared to vehicle-treated tumors (*P* < 0.001 and *P* = 0.001, respectively). Inhibition of aurora B kinase activity did not differ between the combination treatment with low-dose AMG 900 and vorinostat and high-dose AMG 900 alone (*P* = 0.806).

## Discussion

Tumor cell resistance and dose-limiting toxicities frequently result in administration of molecularly targeted agents below the efficacy threshold in patients. The efficacy of treatment could be increased by rationally combining antitumor therapies. Previously, we applied analysis of functional annotation (AFA) to analyze data from a microarray experiment with VPA- or vorinostat-treated PCA cells that demonstrated HDACI-induced changes in gene expression in these cells 23. These data highlighted multiple pathways that were up- or downregulated by vorinostat and VPA in PCA cells 15. One can envisage pathways downregulated by HDACIs as opportunities for combining treatment modalities that are ineffective in the pathways’ presence. Several genes involved in the mitotic spindle checkpoint were downregulated by HDACI treatment, such as aurora kinase inhibitors, pointing to combinations of HDACIs with mitotic spindle checkpoint inhibitors as a promising anticancer strategy 14. Combination therapy of aurora kinase inhibitors with HDACIs had promising results in preclinical experiments with blood cancers 28,29; such combinations have not been assessed in solid tumors. For this purpose, we successfully combined AMG 900, a pan-aurora kinase inhibitor, with HDACIs VPA and vorinostat in PCA cells in this study. AMG 900 is currently being tested in phase I clinical trials in patients with advanced solid tumors and in patients with acute leukemias 30; vorinostat is FDA approved with a primary indication for cutaneous T-cell lymphoma.

Our data indicate that combining aurora kinase inhibitors with HDACIs yields additive and even synergistic effects in inhibiting growth of both androgen-dependent (LNCaP) and androgen-independent (DU-145, PC3) PCA cell lines. We propose that multiple factors contribute to the observed synergy: 1 HDACIs target cells in different stages of the cell cycle (interphase) than AMG 900 (mitosis) 11,31. It is conceivable that cells which escape targeting by AMG 900 and progress through mitosis would be inhibited by HDACIs; 2 HDACIs may complement the action of the aurora kinase inhibitor by directly upregulating expression of genes that trigger the apoptotic/senescence pathway, such as p21, p27, and Bcl-2 28,32; 3 HDACIs also target aurora kinases but from a transcriptional angle instead of by direct catalytic inhibition, therefore the aurora kinase pathway is targeted more effectively with combination therapy 33–35; and 4 our data indicate that combinations of HDACIs and AMG 900 could trigger senescense. Senescence is known to be induced by activation of p21/Cip1 via p53 24. However, AMG 900 induces p21/Cip1 proteins in LNCaP, which has a wild-type p53, and PC3 and DU-145 cells, which are p53 null and mutant, respectively. This indicates that p21 is induced independently of p53. In line with this observation, HDACIs are known to induce p21 and senescence independently of the p53-p21 pathway 32,36,37. Intriguingly, the antitumor effect of AMG 900, enhanced with HDACIs, was more pronounced in clonogenic assays than in MTS assays. In line with previous studies with aurora kinase inhibitors, we therefore hypothesized endoreduplication to be the mechanism of action 10,11,38. Though this process inexorably leads to cell death, it may, prior to that point, leave mitochondrial activity unaffected. Our cell cycle and microscopic imaging analysis results demonstrated an increase in polyploidy and senescence after AMG 900 treatment, supporting this theory.

Consistent with our in vitro observations, tumor growth rates were lower in mice with a DU-145 xenograft model that were treated with a combination of low-dose AMG 900 and vorinostat, than in comparable mice treated with each individual agent at low doses. Growth rates in mice given combination treatment were equivalent, however, to those found in mice treated with high-dose AMG 900 alone. Prior research noted that AMG 900 blocks phosphorylated histone H3 in a dose-dependent manner in multiple human tumor xenograft models 11. Pharmacodynamic analysis in our study demonstrated that low-dose AMG 900 in combination with low-dose vorinostat blocks phosphorylated histone H3 with efficacy similar to that of high-dose AMG 900.

In conclusion, combination treatment of low-dose AMG 900 with HDACIs could prove to be a viable mode of therapy in solid tumors such as PCA. Clinically, a regimen combining lower concentrations of HDACIs and AMG 900 could thereby yield 1) increased efficacy through synergistic or additive mechanisms, and/or 2) decreased toxicity. As VPA, vorinostat and AMG 900 differ in dose-limiting toxicities, the combination is not expected to severely increase the odds of a dose-limiting toxicity 30,39–41. Therefore, this combination of targeted therapies could be a candidate for clinical trials as forward-seeking translational research aimed at improving clinical outcomes in cancer patients.

## References

[1] Tannock IF, de Wit R, Berry WR, Horti J, Pluzanska A, Chi KN (2004). Docetaxel plus prednisone or mitoxantrone plus prednisone for advanced prostate cancer. N. Engl. J. Med.

[2] de Bono JS, Oudard S, Ozguroglu M, Hansen S, Machiels JP, Kocak I (2010). Prednisone plus cabazitaxel or mitoxantrone for metastatic castration-resistant prostate cancer progressing after docetaxel treatment: a randomised open-label trial. Lancet.

[3] Siegel R, Naishadham D, Jemal A (2013). Cancer statistics, 2013. CA Cancer J. Clin.

[4] Hilton JF, Shapiro GI (2014). Aurora kinase inhibition as an anticancer strategy. J. Clin. Oncol.

[5] Das K, Lorena PD, Ng LK, Shen L, Lim D, Siow WY (2010). Aurora-A expression, hormone receptor status and clinical outcome in hormone related cancers. Pathology.

[6] Carmena M, Earnshaw WC (2003). The cellular geography of aurora kinases. Nat. Rev. Mol. Cell Biol.

[7] Sardon T, Peset I, Petrova B, Vernos I (2008). Dissecting the role of Aurora A during spindle assembly. EMBO J.

[8] Perez-Cadahia B, Drobic B, Davie JR (2009). H3 phosphorylation: dual role in mitosis and interphase. Biochem. Cell Biol.

[9] Chieffi P, Cozzolino L, Kisslinger A, Libertini S, Staibano S, Mansueto G (2006). Aurora B expression directly correlates with prostate cancer malignancy and influence prostate cell proliferation. Prostate.

[10] Girdler F, Gascoigne KE, Eyers PA, Hartmuth S, Crafter C, Foote KM (2006). Validating Aurora B as an anti-cancer drug target. J. Cell Sci.

[11] Payton M, Bush TL, Chung G, Ziegler B, Eden P, McElroy P (2010). Preclinical evaluation of AMG 900, a novel potent and highly selective pan-aurora kinase inhibitor with activity in taxane-resistant tumor cell lines. Cancer Res.

[12] Yang H, Burke T, Dempsey J, Diaz B, Collins E, Toth J (2005). Mitotic requirement for aurora A kinase is bypassed in the absence of aurora B kinase. FEBS Lett.

[13] Lee EC, Frolov A, Li R, Ayala G, Greenberg NM (2006). Targeting Aurora kinases for the treatment of prostate cancer. Cancer Res.

[14] Kachhap SK, Rosmus N, Collis SJ, Kortenhorst MS, Wissing MD, Hedayati M (2010). Downregulation of homologous recombination DNA repair genes by HDAC inhibition in prostate cancer is mediated through the E2F1 transcription factor. PLoS One.

[15] Kortenhorst MS, Wissing MD, Rodriguez R, Kachhap SK, Jans JJ, Van der Groep P (2013). Analysis of the genomic response of human prostate cancer cells to histone deacetylase inhibitors. Epigenetics.

[16] Wissing MD, Mendonca J, Kortenhorst MS, Kaelber NS, Gonzalez M, Kim E (2013). Targeting prostate cancer cell lines with polo-like kinase 1 inhibitors as a single agent and in combination with histone deacetylase inhibitors. FASEB J.

[17] Abbas A, Gupta S (2008). The role of histone deacetylases in prostate cancer. Epigenetics.

[18] Shabbeer S, Kortenhorst MS, Kachhap S, Galloway N, Rodriguez R, Carducci MA (2007). Multiple molecular pathways explain the anti-proliferative effect of valproic acid on prostate cancer cells in vitro and in vivo. Prostate.

[19] Hwang JJ, Kim YS, Kim MJ, Kim DE, Jeong IG, Kim CS (2010). Histone deacetylase inhibitor potentiates anticancer effect of docetaxel via modulation of Bcl-2 family proteins and tubulin in hormone refractory prostate cancer cells. J. Urol.

[20] Bots M, Johnstone RW (2009). Rational combinations using HDAC inhibitors. Clin. Cancer Res.

[21] Chou TC, Talalay P (1983). Analysis of combined drug effects: a new look at a very old problem. Trends Pharmacol. Sci.

[22] Chou TC (2010). Drug combination studies and their synergy quantification using the Chou-Talalay method. Cancer Res.

[23] Kortenhorst MS, Zahurak M, Shabbeer S, Kachhap S, Galloway N, Parmigiani G (2008). A multiple-loop, double-cube microarray design applied to prostate cancer cell lines with variable sensitivity to histone deacetylase inhibitors. Clin. Cancer Res.

[24] Roninson IB (2002). Oncogenic functions of tumour suppressor p21(Waf1/Cip1/Sdi1): association with cell senescence and tumour-promoting activities of stromal fibroblasts. Cancer Lett.

[25] Xu WS, Perez G, Ngo L, Gui CY, Marks PA (2005). Induction of polyploidy by histone deacetylase inhibitor: a pathway for antitumor effects. Cancer Res.

[26] Wei Y, Mizzen CA, Cook RG, Gorovsky MA, Allis CD (1998). Phosphorylation of histone H3 at serine 10 is correlated with chromosome condensation during mitosis and meiosis in Tetrahymena. Proc. Natl. Acad. Sci. USA.

[27] Butler LM, Agus DB, Scher HI, Higgins B, Rose A, Cordon-Cardo C (2000). Suberoylanilide hydroxamic acid, an inhibitor of histone deacetylase, suppresses the growth of prostate cancer cells in vitro and in vivo. Cancer Res.

[28] Kretzner L, Scuto A, Dino PM, Kowolik CM, Wu J, Ventura P (2011). Combining histone deacetylase inhibitor vorinostat with aurora kinase inhibitors enhances lymphoma cell killing with repression of c-Myc, hTERT, and microRNA levels. Cancer Res.

[29] Fiskus W, Wang Y, Joshi R, Rao R, Yang Y, Chen J (2008). Cotreatment with vorinostat enhances activity of MK-0457 (VX-680) against acute and chronic myelogenous leukemia cells. Clin. Cancer Res.

[30] Carducci MA, Shaheen MF, Paller CJ, Bauman JE, Azad NS, Shubhakar P (2012). First-in-human study of AMG 900, an oral pan-Aurora kinase inhibitor, in adult patients (pts) with advanced solid tumors. J. Clin. Oncol.

[31] Richon VM, Sandhoff TW, Rifkind RA, Marks PA (2000). Histone deacetylase inhibitor selectively induces p21WAF1 expression and gene-associated histone acetylation. Proc. Natl. Acad. Sci. USA.

[32] DiBernardo G, Squillaro T, Dell'Aversana C, Miceli M, Cipollaro M, Cascino A (2009). Histone deacetylase inhibitors promote apoptosis and senescence in human mesenchymal stem cells. Stem Cells Dev.

[33] Gully CP, Velazquez-Torres G, Shin JH, Fuentes-Mattei E, Wang E, Carlock C (2012). Aurora B kinase phosphorylates and instigates degradation of p53. Proc. Natl. Acad. Sci. USA.

[34] Lorenzo C, Liao Q, Hardwicke MA, Ducommun B (2009). Pharmacological inhibition of aurora-A but not aurora-B impairs interphase microtubule dynamics. Cell Cycle.

[35] Fadri-Moskwik M, Weiderhold KN, Deeraksa A, Chuang C, Pan J, Lin SH (2012). Aurora B is regulated by acetylation/deacetylation during mitosis in prostate cancer cells. FASEB J.

[36] Blagosklonny MV, Robey R, Sackett DL, Du L, Traganos F, Darzynkiewicz Z (2002). Histone deacetylase inhibitors all induce p21 but differentially cause tubulin acetylation, mitotic arrest, and cytotoxicity. Mol. Cancer Ther.

[37] Gediya LK, Belosay A, Khandelwal A, Purushottamachar P, Njar VC (2008). Improved synthesis of histone deacetylase inhibitors (HDIs) (MS-275 and CI-994) and inhibitory effects of HDIs alone or in combination with RAMBAs or retinoids on growth of human LNCaP prostate cancer cells and tumor xenografts. Bioorg. Med. Chem.

[38] Weaver BA, Cleveland DW (2005). Decoding the links between mitosis, cancer, and chemotherapy: the mitotic checkpoint, adaptation, and cell death. Cancer Cell.

[39] Atmaca A, Al-Batran SE, Maurer A, Neumann A, Heinzel T, Hentsch B (2007). Valproic acid (VPA) in patients with refractory advanced cancer: a dose escalating phase I clinical trial. Br. J. Cancer.

[40] Bruserud O, Stapnes C, Ersvaer E, Gjertsen BT, Ryningen A (2007). Histone deacetylase inhibitors in cancer treatment: a review of the clinical toxicity and the modulation of gene expression in cancer cell. Curr. Pharm. Biotechnol.

[41] Bradley D, Rathkopf D, Dunn R, Stadler WM, Liu G, Smith DC (2009). Vorinostat in advanced prostate cancer patients progressing on prior chemotherapy (National Cancer Institute Trial 6862): trial results and interleukin-6 analysis: a study by the Department of Defense Prostate Cancer Clinical Trial Consortium and University of Chicago Phase 2 Consortium. Cancer.

