# Supplementary material for: Combining the pan-aurora kinase inhibitor AMG 900 with histone deacetylase inhibitors enhances antitumor activity in prostate cancer
Source: Cancer Med. 2014 Jul 3;3(5):1322–35. doi: 10.1002/cam4.289 (PMC4302682; doi:10.1002/cam4.289)
Supplement: Supplementary file 1 — Data S1. Supplementary data. [file cam40003-1322-SD1.doc]

**Supplementary Data**


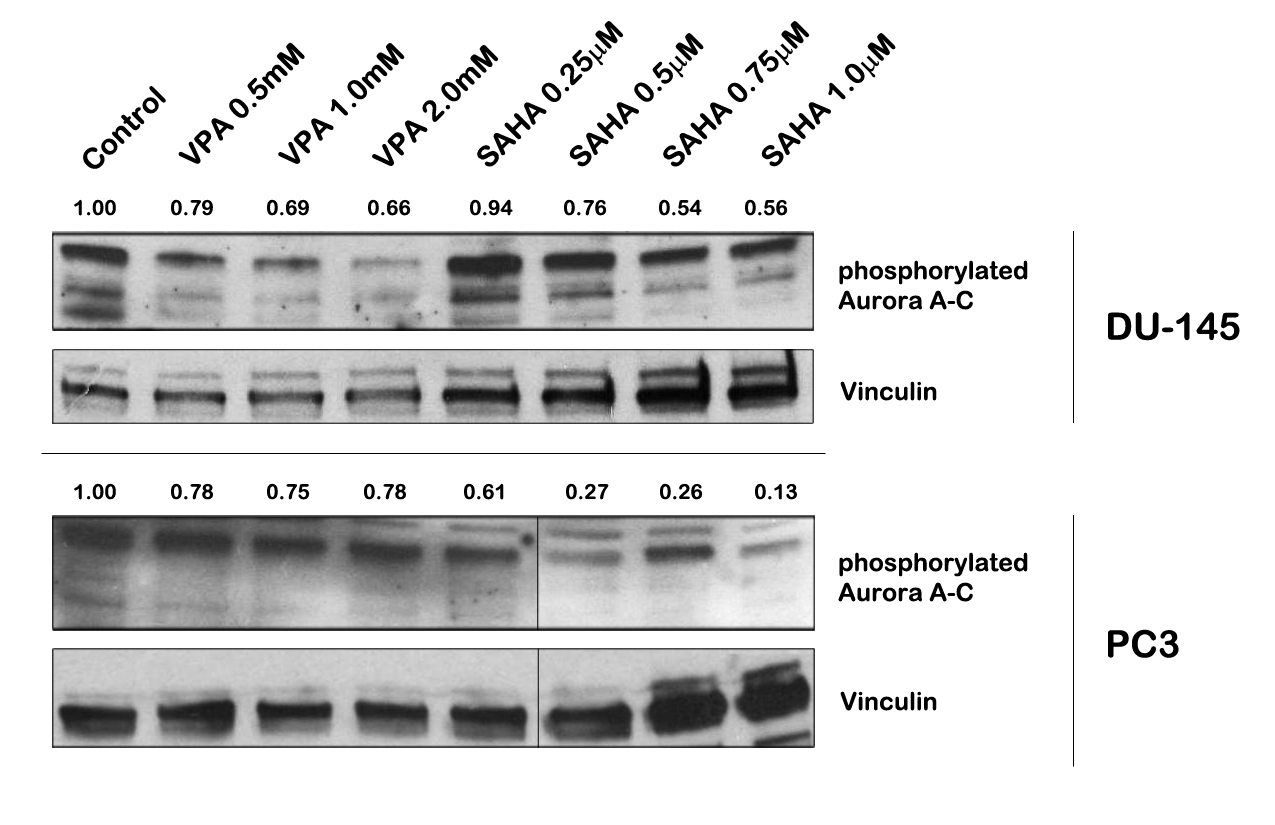


**Supplementary Figure 1.** The HDACIs VPA and vorinostat (SAHA) inhibit expression of phosphorylated aurora A-C of PCA cells at low millimolar or micromolar concentrations, respectively, as depicted by a Western blot for phosphorylated aurora A (48 kDa), B (40 kDa) and C (35 kDa).

|  |  |  |  |  |  |  |  |  |  |  |  |
| --- | --- | --- | --- | --- | --- | --- | --- | --- | --- | --- | --- |
|  | **MTS assays** | | | |  |  | **Clonogenic assays** | | | |  |
|  |  |  |  |  |  |  |  |  |  |  |  |
|  |  | **CI** | | |  |  |  | **CI** | | |  |
|  | **Drug combination** | **DU145** | **PC3** | **LNCaP** |  |  | **Drug combination** | **DU145** | **PC3** | **LNCaP** |  |
|  | Ai 1 nM + VPA 1 mM | 0,796 | ≥1 | 0,848 |  |  | Ai 1 nM + VPA 1 mM | 0,186 | 0,260 | 0,765 |  |
|  | Ai 5 nM + VPA 1 mM | ≥1 | ≥1 | ≥1 |  |  | Ai 5 nM + VPA 1 mM | 0,806 | 0,901 | ≥1 |  |
|  | Ai 1 nM + VPA 1.5 mM | 0,777 | ≥1 | 0,851 |  |  | Ai 1 nM + VPA 1.5 mM | 0,171 | 0,252 | ≥1 |  |
|  | Ai 5 nM + VPA 1.5 mM | ≥1 | ≥1 | ≥1 |  |  | Ai 5 nM + VPA 1.5 mM | 0,869 | 0,998 | ≥1 |  |
|  |  |  |  |  |  |  |  |  |  |  |  |
|  | Ai 1 nM + SAHA 0.5 µM | ≥1 | ≥1 | ≥1 |  |  | Ai 1 nM + SAHA 0.5 µM | 0,340 | 0,583 | 0,593 |  |
|  | Ai 5 nM + SAHA 0.5 µM | ≥1 | ≥1 | ≥1 |  |  | Ai 5 nM + SAHA 0.5 µM | ≥1 | ≥1 | 0,936 |  |
|  | Ai 1 nM + SAHA 1 µM | ≥1 | 0,375 | ≥1 |  |  | Ai 1 nM + SAHA 1 µM | 0,538 | 0,809 | 0,798 |  |
|  | Ai 5 nM + SAHA 1 µM | ≥1 | 0,558 | ≥1 |  |  | Ai 5 nM + SAHA 1 µM | 0,951 | ≥1 | ≥1 |  |
|  |  |  |  |  |  |  |  |  |  |  |  |

**Supplementary Table 1**. Combination index (CI) values as determined by CalcuSyn software. MTS (left) and clonogenic (right) assays were performed in PCA cell lines treated with AMG 900, HDACIs or a combination of inhibitors (Figure 2). CalcuSyn was used to assess whether combination treatments accomplished a synergistic inhibitory effect in PCA cells compared to single agent treatment. CI-values <0.9 indicate a moderately synergistic effect, <0.7 a synergistic effect, <0.3 a strongly synergistic effect, and <0.1 a very strongly synergistic effect. A CI-value ≥0.9 indicates no established synergy in this combination treatment compared to single agent treatment. Ai, AMG 900; SAHA, vorinostat.

**
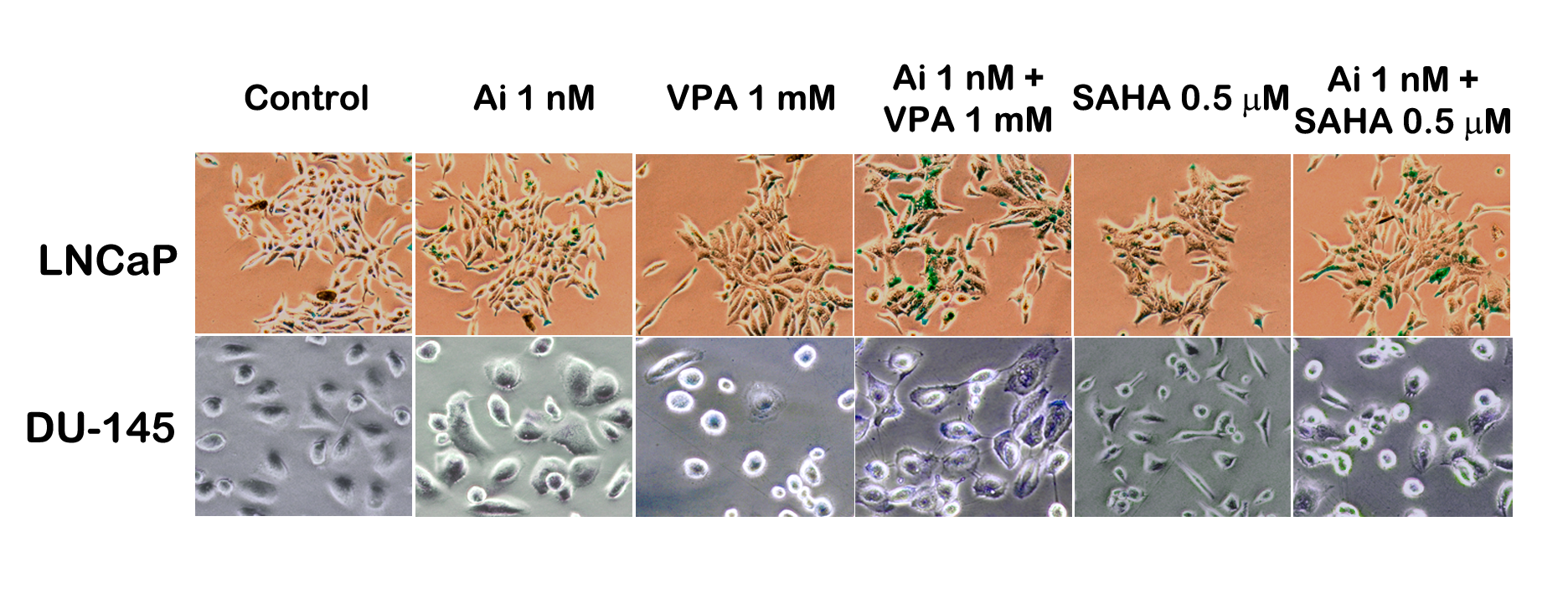
**

**Supplementary Figure 2.** AMG 900 combined with HDACIs VPA or vorinostat (SAHA) increases cellular senescence in PCA cell lines compared to single agent use. Representative images of DU-145 and LNCaP cells after performing an SA β-galactosidase assay. Blue cells are SA β-galactosidase positive cells, indicating cellular senescence. An orange filter was applied in the images with LNCaP cells to improve visibility of SA β-galactosidase in LNCaP cells. Ai, AMG 900.

**Supplementary *in vivo* results**

**Design summary**

This experiment was designed to assess combinations of the aurora kinase inhibitor AMG 900 and the HDACI vorinostat (SAHA) for anti-tumor effects *in vivo*. Prostate cancer cells (DU-145) were injected into NOD/SCID mice. When average tumor volume was above 200 mm^3^, mice were evenly distributed into six treatment groups: control, AMG 3.75 mg/kg, AMG 7.5 mg/kg, SAHA, AMG 3.75 mg/kg plus SAHA and AMG 7.5 mg/kg plus SAHA. Tumor size and weight measurements were taken on days 1, 5, 8, 12, 15, 19, 22, 26 and 29. Analyses assessed the low dose AMG/SAHA combinations for similar efficacy to the single high dose and high dose combinations. Data was analyzed using the random intercept hierarchical linear model, as described in the manuscript.

**Results**

Data were complete for all mice with the exception of the last data point for one mouse in the AMG 7.5 plus SAHA group. The sample size was eight in the control group and nine in all other groups. Supplementary Figures 2 and 3 are line plots of the log tumor volumes and log ratio tumor volumes for each mouse over time. Using the random intercept model described in the methods, the overall effects for time, group, and the time by group interaction were all significant, Table 2. The significant time by group interaction indicates that the growth rates were significantly different between the treatment groups.

**Table 2. Tests of fixed effects for tumor volume**

| **Effect** | **Numerator DF** | **Denominator DF** | **F value** | **P value** |
| --- | --- | --- | --- | --- |
| group | 5 | 59.4 | 3.08 | 0.015 |
| time | 1 | 364 | 920.38 | <.0001 |
| group*time | 5 | 364 | 3.96 | <.0001 |

Model estimates of the mean final log ratio in each group are shown in Table 3. Treatment groups with significantly smaller mean log ratios than the control included: AMG7.5 (p=0.01), AMG 3.75+SAHA (p=0.01), and AMG 7.5+SAHA (p=0.001). The final log ratio in the high dose AMG group, was lower than the low dose AMG, but this difference was not significant, p=0.19.

**Table 3.** **Estimates of mean final log ratio tumor volume by group**

| **Effect** | **Estimate** | **95% Confidence Interval** |
| --- | --- | --- |
| Control | 1.3614 | (1.1299, 1.5929) |
| AMG 3.75 | 1.1590 | (0.9407, 1.3772) |
| AMG 7.5 | 0.9535 | (0.7352, 1.1718) |
| SAHA | 1.1443 | (0.926, 1.3626) |
| AMG 3.75 + SAHA | 0.9389 | (0.7207, 1.1572) |
| AMG 7.5 + SAHA | 0.8147 | (0.5955, 1.0338) |

Estimates of the growth rate (i.e. slopes) for each treatment group are shown in Table 4. Treatment groups with significantly slower growth rates than the control included: AMG7.5 (p=0.02), AMG 3.75+SAHA (p=0.014), and AMG 7.5+SAHA (p=0.036) (Table 5). The rates in the single drug low dose AMG and SAHA groups were higher than the low dose AMG+SAHA combination, p=0.003 and p=0.008 respectively. The high dose AMG growth rate was also significantly slower than the low dose AMG rate, p=0.005.

**Table 4. Estimates of growth rates** by treatment group

| **Effect** | **Estimate** | **95% Confidence Interval** |
| --- | --- | --- |
| Control | 0.04242 | (0.03614, 0.04871) |
| AMG 3.75 | 0.04451 | (0.03858, 0.05044) |
| AMG 7.5 | 0.03245 | (0.02652, 0.03837) |
| SAHA | 0.04297 | (0.03704, 0.0489) |
| AMG 3.75+SAHA | 0.03155 | (0.02562, 0.03748) |
| AMG7.5+SAHA | 0.03309 | (0.02705, 0.03912) |

**Table 5. P-values comparing differences in tumor growth rates between treatment groups**

| **Compare...** | **to...** | **Growth rate** | |
| --- | --- | --- | --- |
| Control | Ai 3.75 nM | 0.64 |  |
| Control | Ai 7.5 nM | 0.02 | * |
| Control | SAHA | 0.90 |  |
| Control | Ai 3.75 nM + SAHA | 0.01 | * |
| Control | Ai 7.5 nM + SAHA | 0.04 | * |
| Ai 3.75 nM | Ai 7.5 nM | 0.01 | * |
| Ai 3.75 nM + SAHA | Ai 3.75 nM | 0.00 | * |
| Ai 3.75 nM + SAHA | SAHA | 0.01 | * |
| Ai 3.75 nM + SAHA | Ai 7.5 nM | 0.83 |  |
| Ai 3.75 nM + SAHA | Ai 7.5 nM + SAHA | 0.72 |  |

*, treatment groups with a significantly different tumor growth rate (p≤0.05). Ai, AMG 900.

The weights of the mice in this study were recorded at the same time tumor volumes were measured and analyzed similarly. Weight loss was of interest in this study as an indicator of toxicity. Supplementary Figures 4 and 5 are line plots of the weights and log ratio weights for each mouse over time. Adjusting for initial weight, by dividing weights on days 5 through 29 by the initial weight, and then taking the log, a random intercept model was fit to determine the effects for time, group, and the time by group interaction. While the overall group effect was not significant (see also Supplementary Figure 6), the effects for time, and the time by group interaction were significant, Table 6. This indicates that the final weights were not different between groups.

**Table 6. Tests of fixed effects for mouse weight**

| **Effect** | **Numerator DF** | **Denominator DF** | **F value** | **P value** |
| --- | --- | --- | --- | --- |
| group | 5 | 63 | 0.75 | 0.5896 |
| time | 1 | 364 | 5.49 | 0.0197 |
| group*time | 5 | 364 | 2.63 | 0.0239 |

Model estimates of the mean final log ratio weight in each group are shown in Table 7. The overall test of differences between these groups was not significant, p=0.59.

**Table 7.** **Estimates of mean final log ratio weight by group**

| **Effect** | **Estimate** | **95% Confidence Interval** |
| --- | --- | --- |
| Control | -0.00426 | (-0.0392, 0.03068) |
| AMG 3.75 | 0.00541 | (-0.0275, 0.03836) |
| AMG 7.5 | -0.01874 | (-0.0517, 0.01421) |
| SAHA | -0.01638 | (-0.0493, 0.01657) |
| AMG 3.75+SAHA | -0.01712 | (-0.0501, 0.01583) |
| AMG7.5+SAHA | -0.03701 | (-0.0701, -0.0039) |


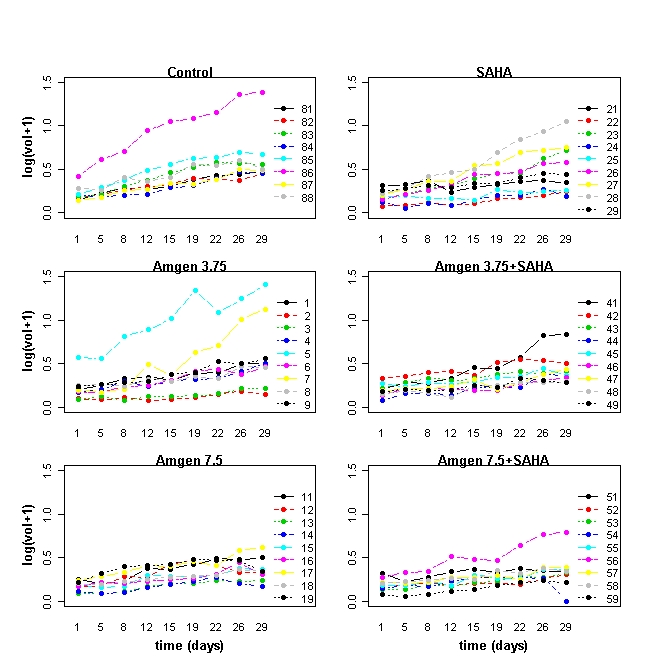


**Supplementary Figure 3.** Line plot of tumor volumes over time for the AMG/SAHA analysis.


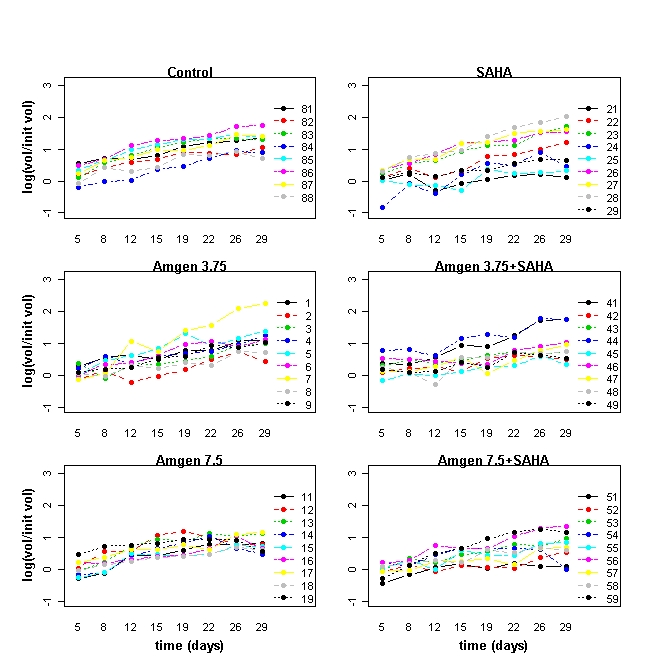


**Supplementary Figure 4.** Line plot of tumor volumes, adjusting for initial volume.


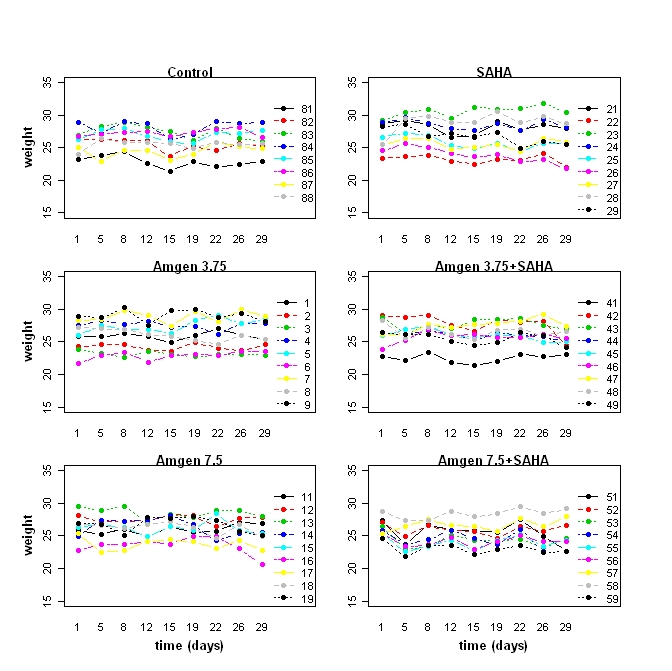


**Supplementary Figure 5.** Line plot of mouse weight (grams) over time for the AMG/SAHA analysis.


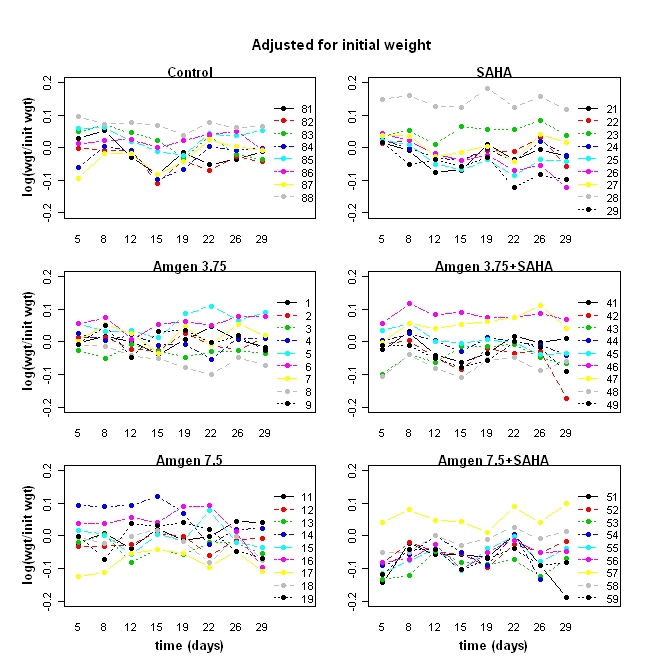


**Supplementary Figure 6.** Line plot of mouse weight (grams), adjusting for initial volume over time for the AMG/SAHA analysis.


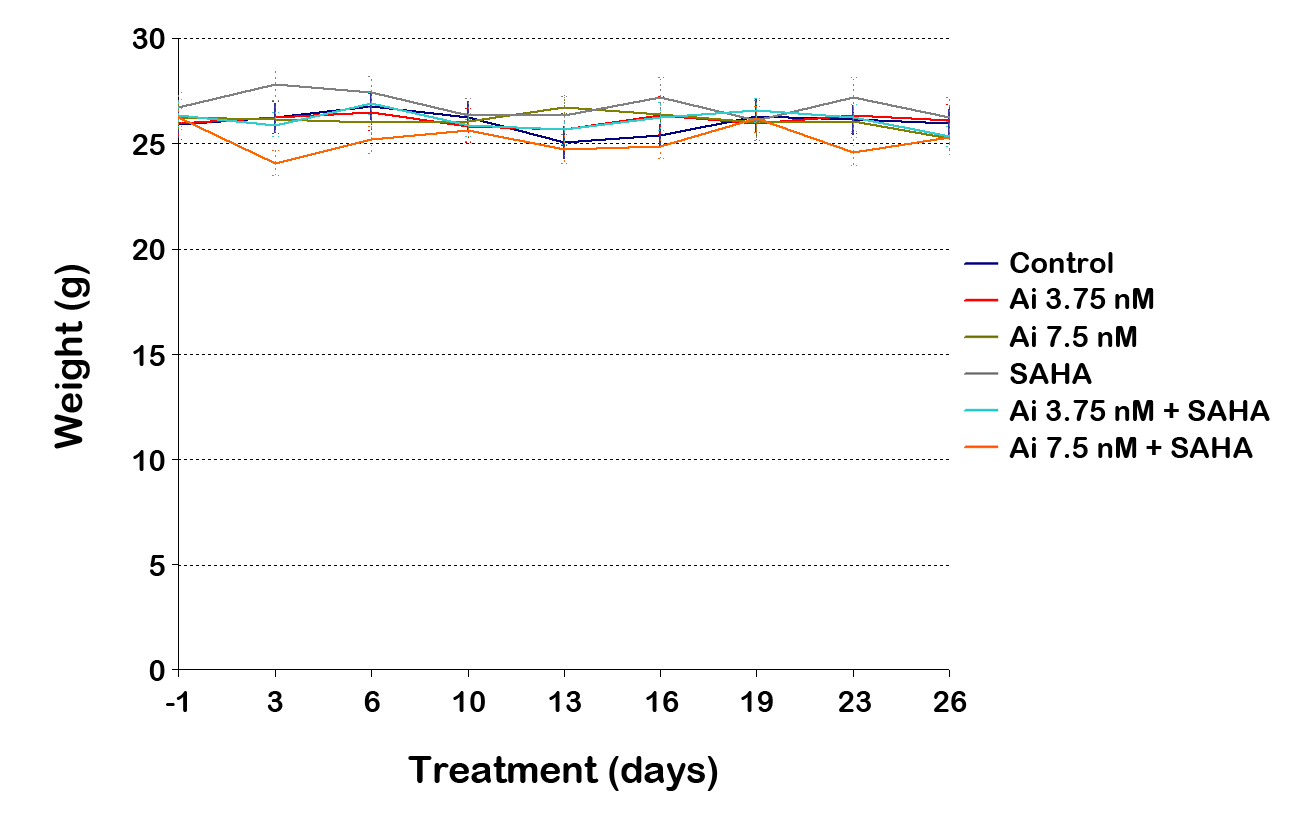


**Supplementary Figure 7.** Combination treatment with AMG 900 and SAHA does not result in significant weight loss *in vivo* (p>0.05). Although the group of mice treated with 7.5 nM and SAHA initially lost weight compared to the other groups (<10%), the average weight of this group stabilized within a week. No other weight changes were evident. Ai, AMG 900.
